# Improved Tuning Fork for Terahertz Quartz-Enhanced Photoacoustic Spectroscopy

**DOI:** 10.3390/s16040439

**Published:** 2016-03-25

**Authors:** Angelo Sampaolo, Pietro Patimisco, Marilena Giglio, Miriam S. Vitiello, Harvey E. Beere, David A. Ritchie, Gaetano Scamarcio, Frank K. Tittel, Vincenzo Spagnolo

**Affiliations:** 1 Dipartimento Interateneo di Fisica, Università degli studi di Bari Aldo Moro e Politecnico di Bari, Via Amendola 173, Bari I-70126, Italy; angelo.sampaolo@uniba.it (A.S.); pietro.patimisco@uniba.it (P.P.); marilena.giglio@uniba.it (M.G.); gaetano.scamarcio@uniba.it (G.S.); 2 Department of Electrical and Computer Engineering, Rice University, 6100 Main Street, Houston, TX 77005, USA; fkt@rice.edu; 3 NEST, CNR-Istituto Nanoscienze and Scuola Normale Superiore, Piazza San Silvestro 12, Pisa I-56127, Italy; miriam.vitiello@sns.it; 4 Cavendish Laboratory, University of Cambridge, J. J. Thomson Avenue, Cambridge CB3 0HE, UK; heb1000@cam.ac.uk (H.E.B.); dar11@cam.ac.uk (D.A.R.)

**Keywords:** quartz enhanced photoacoustic spectroscopy, quartz tuning fork, gas sensing, THz spectroscopy, quantum cascade laser

## Abstract

We report on a quartz-enhanced photoacoustic (QEPAS) sensor for methanol (CH_3_OH) detection employing a novel quartz tuning fork (QTF), specifically designed to enhance the QEPAS sensing performance in the terahertz (THz) spectral range. A discussion of the QTF properties in terms of resonance frequency, quality factor and acousto-electric transduction efficiency as a function of prong sizes and spacing between the QTF prongs is presented. The QTF was employed in a QEPAS sensor system using a 3.93 THz quantum cascade laser as the excitation source in resonance with a CH_3_OH rotational absorption line located at 131.054 cm^−1^. A minimum detection limit of 160 ppb in 30 s integration time, corresponding to a normalized noise equivalent absorption NNEA = 3.75 × 10^−11^ cm^−1^W/Hz^½^, was achieved, representing a nearly one-order-of-magnitude improvement with respect to previous reports.

## 1. Introduction

Spectroscopic techniques for trace gas sensing and monitoring have shown great potential for non-invasive chemical analysis requiring high sensitivity and selectivity. While trace gas detection in the near-infrared (IR) and mid-IR ranges demonstrated excellent performance, the use of far-IR or terahertz (THz) radiation for sensing purposes is still underdeveloped. In the wavelength range from 50 μm to 3 mm wavelengths (0.1–6 THz), numerous gas molecules have well-defined spectral THz “fingerprints” absorptions, due to strong rotational transitions between molecular energetic levels. Hence, the THz spectral region can provide higher detection selectivities compared to those provided by the characteristic ro-vibrational complex structures in the mid-IR range. A number of THz optical systems have been proposed for gas sensing and spectroscopy. Terahertz Time-Domain Spectroscopy (THz-TDS) is based on frequency conversion employing nonlinear optics and femtosecond laser pulses for the generation and detection of THz radiation [1]. Spectral measurements over a very broad bandwidth in the THz range can be performed using THz-TDS, allowing spectroscopic investigation of a large number of gases, such as water vapor, methyl chloride and nitrous oxide [2,3,4]. However, THz-TDS is strongly limited by the available μW-range output optical powers. Furthermore, such a technique usually provides a limited control of the selected frequency bandwidth. The sensitivity of THz gas spectroscopy can be enhanced by increasing the available optical power. So far, THz quantum cascade lasers (QCLs) represent an attractive solution in the far-IR spectral region in terms of output power [5,6] and spectral purity [7]. THz QCLs can provide single-mode emission with output powers of up to 138 mW in continuous wave (cw) operation at cryogenic temperatures [8]. Wavelength modulation spectroscopy, employing liquid-He-cooled cw-operating QCLs combined with low-noise bolometer detectors, is promising in terms of THz-sensing performances [9,10]. This approach would allow reaching high sensitivity and selectivity, but suffers from complexity and the disadvantage of using cryogenic cooling systems for both generation and detection of THz radiation.

Recently, THz quartz-enhanced photoacoustic (QEPAS) sensors were reported [11,12,13]. QEPAS permits overcoming some issues traditionally associated with THz spectroscopy, such as the use of cryogenic systems for the detection of THz radiation and complex signal analysis processes. QEPAS is based on the detection of acoustic waves produced by the absorbing gas target by means of a piezoelectric quartz tuning fork (QTF) acting as an acousto-electric transducer [11,12,13,14,15,16]. The merits of QEPAS include high detection sensitivity, high selectivity, and fast time response using a compact and relatively low-cost acoustic detection module [16,17,18,19]. Since rotational levels are up to three orders of magnitude faster with respect to mid-IR vibrational levels, the THz spectral range is particularly suitable for the QEPAS technique. Indeed, the fast relaxation times characteristic of THz transitions allow low-pressure operation, providing high QTF resonance Q-factors, and thereby large QEPAS signals [16].

The extension of QEPAS in the THz range was made possible by the realization of a custom-made QTF [11,12] (denoted in this work as C-QTF) having the same geometry as the commercial 32.78 kHz-QTF, but size six times larger, with prong lengths of *L* = 20.0 mm, prong widths of *T* = 1.4 mm and a crystal thickness of *w* = 0.8 mm. The QTF prongs are separated by a ~1 mm gap, required to focus a THz laser beam between the prongs without illuminating them. It is worth noting that the standard QTF structure is optimized for timing purposes and is not ideal for spectroscopic applications.

In a previous study [20], we investigated the electro-elastic properties of a set of six QTFs with different geometries and the objective of determining the optimal design for optoacoustic gas sensing. This study allowed us to identify a novel QTF design (denoted in this work as N-QTF), optimized for THz QEPAS. 

The performance of a QEPAS sensor can be considered in terms of its QEPAS signal (*S*) which can be expressed as *S = KP_0_Qαε*, where *K* represents the QTF efficiency in converting the acoustic pressure wave incident on the internal side of the two prongs into transversal in-plane deflections, *P_0_* is the laser power, *Q* is the QTF quality factor, *α* is the gas absorption coefficient and *ε* is the conversion efficiency of the absorbed optical power into sound. The factor *Q·K* can be used as the figure of merit to compare the sensing performance of the two QTFs in a QEPAS sensor, if all other sensor parameters (e.g., laser power, absorption strength, gas pressure and sound conversion efficiency) are kept constant. The N-QTF was used in the same QEPAS platform as previously used with the C-QTF to detect methanol [11,12]. A comparison of the performances between the two THz QEPAS sensor systems shows a sensitivity enhancement of approximately one order of magnitude when using the N-QTF.

## 2. Quartz Tuning Fork Design and Implementation

To investigate the influence of QTF sizes on the acousto-electric transduction efficiency, *i.e.*, the conversion efficiency of the acoustic wave in piezoelectric charges, we selected three QTF parameters that directly influence this process: (i) the QTF resonance frequency *f*; (ii) its quality factor *Q*; and (iii) the spacing *s* between the two prongs [20].

In a typical QEPAS sensor, the laser modulation frequency *f_L_* is set at one of the QTF resonance frequencies or the related sub-harmonics [16]. To ensure that the gas non-radiative energy transfer, which generates acoustic waves, follows the fast modulation *f_L_* of the incident laser radiation efficiently, the condition *f_L_ << 1/2πτ* must be satisfied [21], where *τ* is the time constant of non-radiative gas relaxation processes. The time constant *τ* depends on the specific gas carrier as well as intermolecular interactions and typically falls in the microseconds range [22]. Hence, the QTF resonance frequency should be reduced with respect to the standard 32.78 kHz QTF, in order to approach the typical relaxation time of gases, resulting in more efficient sound wave generation and providing an increase of the QEPAS signal. Recently, a QTF with a resonance frequency of 30.72 kHz was employed in a QEPAS sensor operating in the near-IR spectral region. Its performance was compared with the same QEPAS sensor architecture with a standard 32.78 kHz QTF, and an increase of the QEPAS signal by a factor of 1.5 was measured [23]. 

The resonance frequencies are defined by the QTF’s material properties and its geometry and can be calculated by considering one arm of the fork as a cantilever-vibrating prong. In QEPAS spectroscopy, only the QTF symmetric flexural modes, where the tines move in opposite directions, are excited. For these flexural modes, the related resonance frequencies depend on prong sizes as *f~T/L^2^* [20]. For a QTF operating in air at the fundamental flexural mode, the *Q* was shown to be proportional to *Tw/L* [20]. Since the QTF piezoelectric signal is directly proportional to *Q*, this parameter should be as high as possible. 

Even if *f* and *Q* are not influenced by the prong spacing *s*, this parameter plays a crucial role in the acousto-electric transduction efficiency. For a focused laser beam, the decay of incident acoustic wave pressure on the prong depends critically on the distance between the focused laser beam position and the internal prong surface. On the other hand, the larger the laser beam waist, the larger the *s* value must be in order to avoid the power beam profile tails hitting the prongs and thereby introducing a modulated fringe-like background noise. Hence, *s* should be chosen based on the expected beam waist of the laser source to be employed. In a previously reported THz QEPAS sensor [12], the laser beam was focused between a prong gap *s* = 1 mm with a ~430-μm-diameter beam waist and it allowed 100% of the laser light to pass through the employed C-QTF without touching the prongs. This result indicated that a reduction of *s* is feasible. Hence, *s* was decreased to 700 µm in the design of the N-QTF. A reduced prong thickness of *T* = 1 mm (1.4 mm for the C-QTF) allowed a further decrease of the resonance frequency. In addition, the prong length was reduced to *L* = 17 mm from L = 20 mm in order to maintain a high quality factor, Q. The fabrication of the N-QTF was realized by Statek, Orange, CA, starting from a crystal plate of thickness *w* = 250 µm (a standard for commercial QTF resonators).

**Figure 1:**
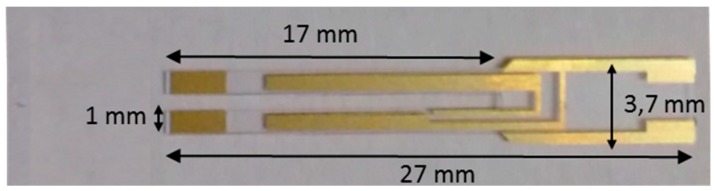
Picture of the N-QTF including the size of the main geometrical parameters.

Figure 1 shows the design of the N-QTF and its main geometrical parameters.

The electrical characterization of the N-QTF was carried out by applying a sinusoidal voltage provided by a wave-function generator. The electrical response of the resonator was processed by a transimpedance amplifier and then demodulated at the same frequency of the voltage excitation by means of a lock-in amplifier. The resonance frequency measured in standard air at a pressure of 10 Torr is *f* = 2871 Hz lower than that measured for the C-QTF (4250 Hz). A quality factor of *Q* = 18,600 was obtained, which is lower than that obtained for the C-QTF (>30,000) with the same experimental conditions. This is mostly due to the smaller crystal thickness of *w* = 250 µm with respect to w = 800 µm for the C-QTF.

## 3. THz QEPAS Sensor Architecture

**Figure 2:**
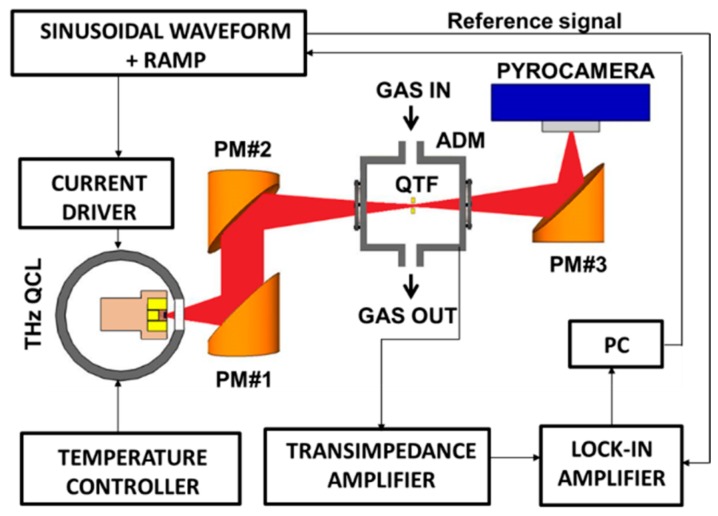
Schematic of the QEPAS trace gas sensor using a THz Quantum Cascade Laser (THz QCL) as the excitation source. PM—Parabolic Mirror; ADM—Acoustic Detection Module; QTF—Quartz Tuning Fork; PC—Personal Computer.

The CH_3_OH QEPAS sensor scheme, similar to the one reported in [11,12], is depicted in Figure 2. We employed the same laser source, which allowed targeting the methanol absorption line located at ν = 3.9289 THz (131.054 cm^−1^) with a line-strength of S = 4.28 × 10^−21^ cm/mol in HITRAN units. 

The THz laser beam was focused between the N-QTF prongs by using two off-axis paraboloidal aluminum reflectors (PM#1 with f-number = 2 and PM#2 with f-number = 5). The N-QTF is fixed on a mounting structure and housed in an acoustic detection module (ADM), with polymethylpentene (TPX) input and output windows. An optical power of 40 μW was measured between the prongs of the QTF by means of a pyroelectric detector (not shown in Figure 2). The THz laser beam exiting the ADM was re-focused onto a pyroelectric camera (Spiricon Pyrocam III-C) by means of an additional aluminum parabolic mirror (PM#3 with f-number = 2). QEPAS measurements were performed by applying a sinusoidal waveform provided by a function generator (Tektronix model AFG3102) at the QTF resonance frequency *f* to the current driver while demodulating the QTF response at *f* by using a lock-in amplifier (Stanford Research Model SR830). Both instruments were controlled by LabView-based software. A slow voltage ramp allows scanning of the THz laser wavelength through the selected methanol absorption line for spectral line-shape acquisition.

**Figure 3:**
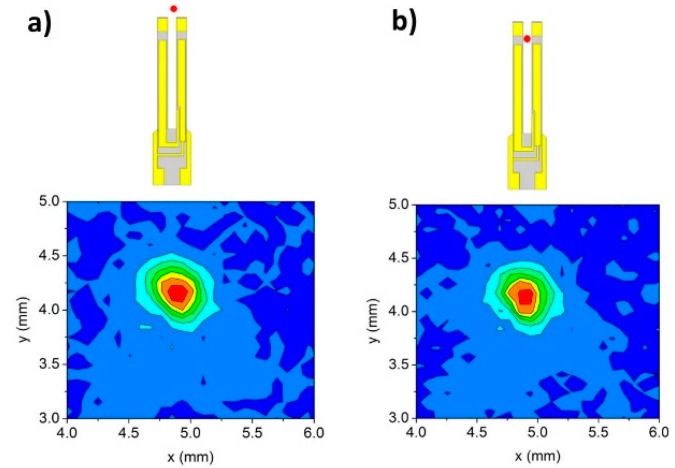
Two-dimensional beam profile of the THz-QCL acquired by means of an IR pyrocamera after mirror PM#3 (see Figure 2) when the beam is focused out of the N-QTF (**a**) or between the two prongs (**b**). Both beam profiles are shown together with an illustration representing the position of the focused THz beam (red spot) with respect to the N-QTF.

Figure 3 depicts the observed two-dimensional (2D) laser beam profiles after mirror PM#3. First the profile is measured with the focused laser beam positioned out of the N-QTF (Figure 3a) and then between the N-QTF prongs (Figure 3b). A comparison between the two beam profiles clearly indicated that the THz beam is not obstructed by the QTF when it is focused between its prongs. The total image intensity was measured by summing all pixel values for both beam profiles, from which we estimated that 96.4% of the light intensity passes between the prongs. Hence, a reduction of the prong spacing from *s* = 1000 μm to *s* = 700 μm should not significantly affect the noise level of the QEPAS signal. 

## 4. THz QEPAS Sensor Calibration and Performance

**Figure 4:**
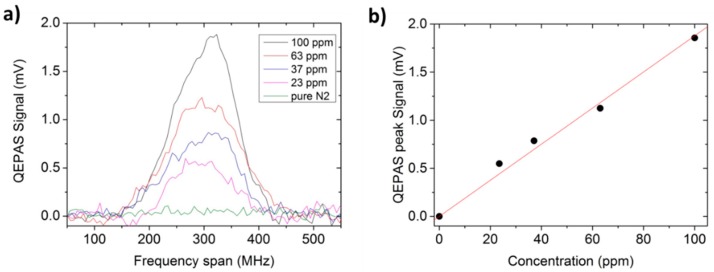
(**a**) QEPAS spectral scans of gas mixture containing different concentrations of methanol in N_2_ at a gas pressure of 10 Torr acquired with 3 s lock-in integration time. The spectral scan obtained for pure N_2_ under the same operating conditions is also depicted. (**b**) Calibration curve (solid red line) obtained from the linear fit of measured QEPAS peak signals (●) *vs.* methanol concentrations.

The sensor calibration was performed by using a trace gas standard generator. Starting from a certified 100 part per million (ppm) CH_3_OH in N_2_ mixture, we produced lower methanol concentrations using pure N_2_ as the diluting gas. A laser modulation amplitude of 600 mV was employed in the QEPAS experiments, similar to those reported in [11,12]. High-resolution QEPAS scans of CH_3_OH:N_2_ calibrated mixtures with different concentrations are shown in Figure 4a, together with a spectral scan acquired when pure N_2_ flows inside the ADM. The lock-in integration time was set to 3 s for all measurements.

The calibration curve shown in Figure 4b was obtained using CH_3_OH concentrations derived from the gas mixture generator and the QEPAS peak signals acquired from the related scans. These results confirm that the QEPAS signal is proportional to the methanol concentration.

**Figure 5:**
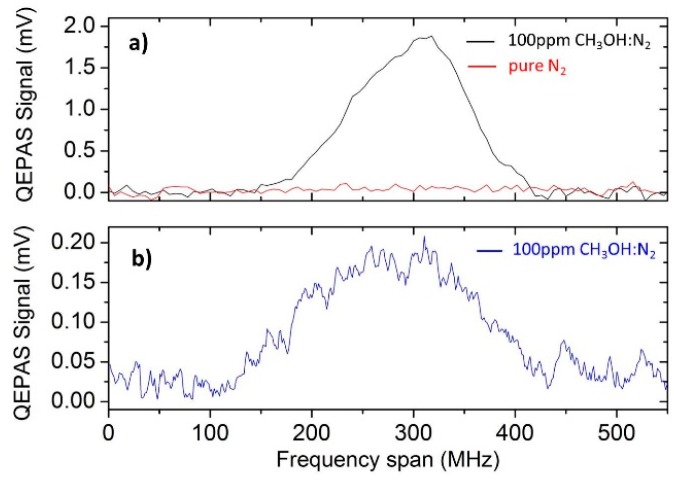
(**a**) Spectral scan of 100 ppm of methanol in N_2_ at a gas pressure of 10 Torr acquired with a 3 s lock-in integration time using the N-QTF. (**b**) Spectral scan of 100 ppm of methanol in N_2_ obtained for the same experimental conditions using the C-QTF with a standard geometry. The lower data sampling in panel (**a**) is due to a faster voltage ramp employed in this work with respect to the measurements reported in [11].

Figure 5 shows a comparison between the QEPAS scan measured for 100 ppm methanol in N_2_ using the N-QTF (Figure 5a) based on the scan obtained at the same concentration for the C-QTF (Figure 5b). In both cases, the QEPAS sensors operated at 10 Torr gas pressure with a lock-in integration time of 3 s. A 1σ minimum detection limit (MDL) using the N-QTF was measured from the analysis of the signal-to-noise ratio and results in an approximately nine-times-better MDL than that previously obtained with the C-QTF. Since the N-QTF is characterized by a lower *Q*, the observed enhancement in the QEPAS sensitivity can be attributed to a reduction of *s*, thus demonstrating its effective influence on the QTF acousto-electric transduction efficiency. The QTF resonance frequency reduction also contributes to the QEPAS signal enhancement. The MDL was 1.7 ppm compared to the previously reported MDL of 15 ppm, while the noise fluctuations (1σ value) recorded with the new QTF are ±30 μV of the same order of those reported in [11] (±25 μV). In principle, a further reduction of the prong spacing can enhance the QEPAS signal even more. However, an increase of the background noise can be also expected, because *s* becomes comparable with the focused laser beam size.

**Figure 6:**
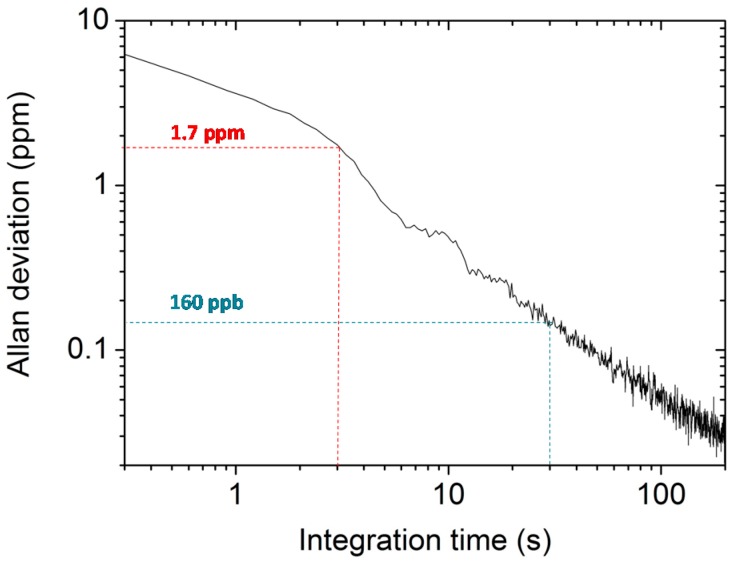
Allan-Werle deviation in ppm as a function of the lock-in integration time for the QEPAS sensor. The curve was calculated by analyzing 120-min-long acquisition periods of the signal measured for pure N_2_ at 10 Torr and setting the lock-in integration time at 100 ms.

An Allan-Werle variance analysis [24] was performed in order to determine the best achievable sensitivity of the THz QEPAS sensor. We measured and averaged the QEPAS signal at zero CH_3_OH concentration (pure N_2_ at 10 Torr) and obtained the Allan-Werle deviation in ppm, shown in Figure 6.

For a 30 s averaging time, a detection sensitivity of 160 part per billion (ppb), corresponding to a normalized noise equivalent absorption NNEA = 3.75 × 10^−11^ cm^−1^W/Hz^½^ (laser power of ~40 μW), was achieved, which represents a new record value for QEPAS trace gas sensing. These results clearly demonstrate that the N-QTF geometry provides better sensor system performances with respect to the standard geometry. An even higher sensitivity can be expected by increasing the QTF crystal thickness *w*; however, chemical etching for a crystal of *w* > 1 mm cannot guarantee sharp edge profiles.

## 5. Conclusions

In this work we reported on a QEPAS THz sensor for CH_3_OH detection, implementing a QTF with a novel geometry. In the new design, the prong width and length were reduced with respect to the previously employed C-QTF with a standard design in order to reduce the resonance frequency while maintaining a high quality factor. We also reduced the prong spacing, enhancing the acousto-electric transduction efficiency. To evaluate the N-QTF performance, we utilized the same QEPAS THz sensor for CH_3_OH detection as reported in [11]. A MDL of 160 ppb at 30 s integration time and a corresponding record of NNEA = 3.75 × 10^−11^ cm^−1^W/Hz^½^ were achieved. The CH_3_OH sensing system performance was enhanced by nearly an order of magnitude with respect to the QEPAS sensor employing a C-QTF with a standard geometry.
